# Supplementary material for: Phenotypic Characterization and Heterogeneity among Modern Clinical Isolates of Acinetobacter baumannii
Source: Microbiol Spectr. 2022 Dec 8;11(1):e03061-22. doi: 10.1128/spectrum.03061-22 (PMC9927488; doi:10.1128/spectrum.03061-22)

**Supplementary Table 1: Natural transformation of several modern isolates and established strains.** Summary of the number of colonies detected after transformation with a plasmid conferring Apramycin resistance and containing *gfp* coding sequence (see Materials and Methods): >50 colonies (+++); 30 – 50 colonies (++); <30 colonies (+); no fluorescent colony detected in the tested conditions (-). Strain resistant to Apramycin before transformation (ApraR). Not determined (Nd).

| Strain | Level of competence | Strain | Level of competence |
|---|---|---|---|
| AB5075-VUB | +++ | AB181-VUB | - |
| DSM30011-VUB | ++ | AB183-VUB | - |
| ATCC17978-VUB | - | AB186-VUB | +++ |
| ATCC19606-VUB | ApraR | AB187-VUB | - |
| AB3-VUB | - | AB188-VUB | Nd |
| AB9-VUB | Nd | AB189-VUB | +++ |
| AB14-VUB | - | AB193-VUB | - |
| AB16-VUB | - | AB194-VUB | Nd |
| AB20-VUB | +++ | AB212-VUB | Nd |
| AB21-VUB | Nd | AB213-VUB | - |
| AB32-VUB | + | AB214-VUB | Nd |
| AB36-VUB | Nd | AB216-VUB | - |
| AB39-VUB | Nd | AB217-VUB | + |
| AB40-VUB | Nd | AB219-VUB | - |
| AB167-VUB | Nd | AB220-VUB | + |
| AB169-VUB | +++ | AB222-VUB | +++ |
| AB171-VUB | Nd | AB224-VUB | Nd |
| AB172-VUB | + | AB226-VUB | - |
| AB173-VUB | - | AB227-VUB | +++ |
| AB175-VUB | + | AB229-VUB | +++ |
| AB176-VUB | +++ | AB231-VUB | Nd |
| AB177-VUB | - | AB232-VUB | +++ |
| AB179-VUB | - | AB233-VUB | Nd |
| AB180-VUB | - | | |

**Supplementary Table 2: Level of capsulation of the clinical isolates and established strains of *A. baumannii*.** The capsulation level was determined by density gradients (see Materials and Methods) and strains were divided to low- (0-0,65 cm), medium- (0,65-1,3 cm) and high-producers (>1,3 cm) of capsule. Strains displaying division into two fractions in different levels are marked with asterisk (*).

| Strain | Level of capsulation | Strain | Level of capsulation |
|---|---|---|---|
| AB5075-VUB | medium | AB181-VUB | medium |
| DSM30011-VUB | low | AB183-VUB | low |
| ATCC17978-VUB | low | AB186-VUB | low |
| ATCC19606-VUB | medium | AB187-VUB | high |
| AB3-VUB | high | AB188-VUB | medium |
| AB9-VUB | high | AB189-VUB | low |
| AB14-VUB | medium | AB193-VUB | high |
| AB16-VUB | high | AB194-VUB | medium |
| AB20-VUB | medium | AB212-VUB | low |
| AB21-VUB | low | AB213-VUB | high |
| AB32-VUB | medium | AB214-VUB | low |
| AB36-VUB | medium | AB216-VUB | low |
| AB39-VUB | medium | AB217-VUB | low |
| AB40-VUB | medium | AB219-VUB | medium |
| AB167-VUB | medium | AB220-VUB | low, medium* |
| AB169-VUB | medium | AB222-VUB | low |
| AB171-VUB | medium | AB224-VUB | medium |
| AB172-VUB | medium | AB226-VUB | medium |
| AB173-VUB | medium | AB227-VUB | medium |
| AB175-VUB | low, medium* | AB229-VUB | medium |
| AB176-VUB | medium | AB231-VUB | low |
| AB177-VUB | medium | AB232-VUB | medium |
| AB179-VUB | medium, high* | AB233-VUB | high |
| AB180-VUB | low | *E. coli* S17 | low |

**Supplementary Figure 1: Corresponding photographs of the macrocolonies of each strain.** The representative photographs of each group is displayed in the Figure 3.

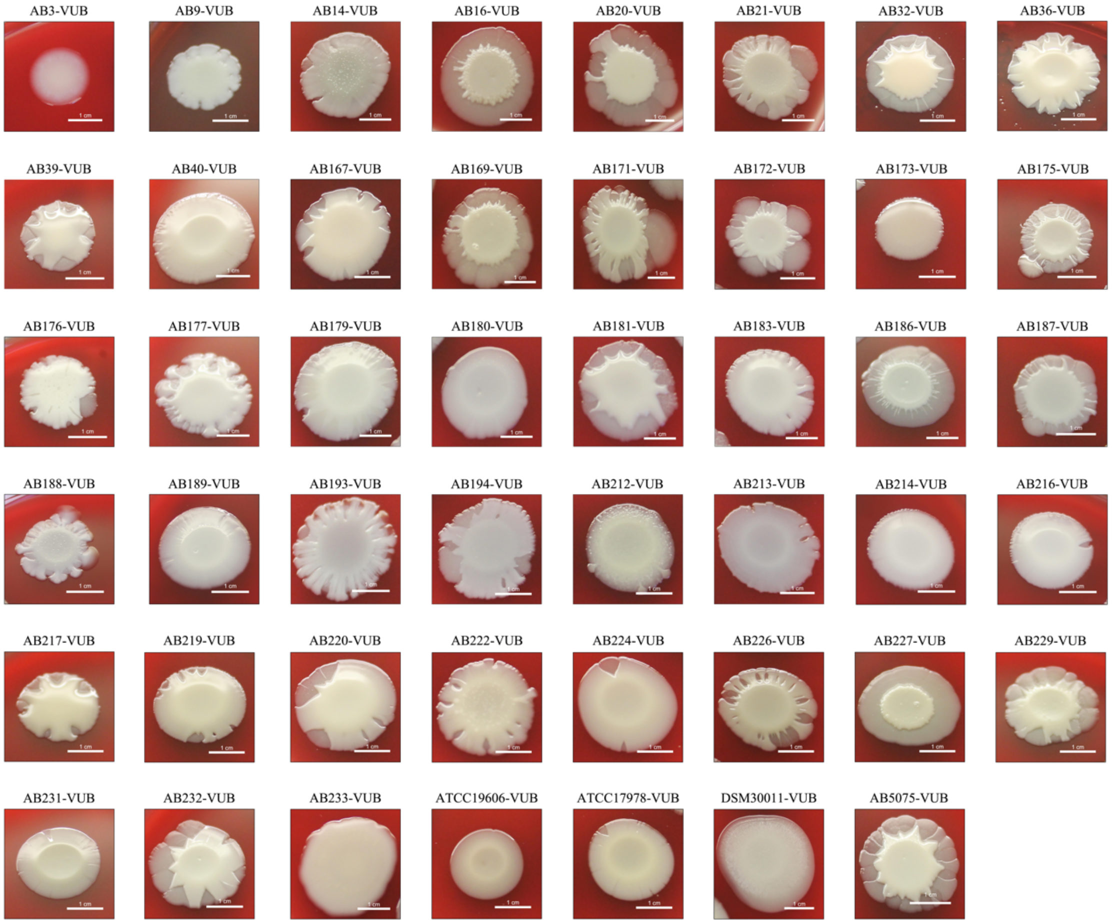

Supplement: Supplemental file 1 — Supplemental material. Download spectrum.03061-22-s0001.pdf, PDF file, 1.8 MB [file spectrum.03061-22-s0001.pdf]
